# Adaptive dosing and platinum–DNA adduct formation in children receiving high-dose carboplatin for the treatment of solid tumours

**DOI:** 10.1038/sj.bjc.6603607

**Published:** 2007-02-13

**Authors:** G J Veal, J Errington, M J Tilby, A D J Pearson, A B M Foot, H McDowell, C Ellershaw, B Pizer, G M Nowell, D G Pearson, A V Boddy

**Affiliations:** 1 Northern Institute for Cancer Research, University of Newcastle upon Tyne, Newcastle upon Tyne, NE2 4HH, UK; 2 Royal Marsden Hospital, Surrey, SM2 5PT, UK; 3 Bristol Royal Hospital for Children, Bristol, BS2 8BJ, UK; 4 Alder Hey Children's Hospital, Liverpool, L12 2AP, UK; 5 United Kingdom Children's Cancer Study Group, Leicester, LE1 6TP, UK; 6 Department of Earth Sciences, Durham University, Durham, DH1 3LE, UK

**Keywords:** carboplatin, therapeutic monitoring, clinical pharmacology, paediatrics, platinum–DNA adducts

## Abstract

A pharmacokinetic–pharmacodynamic study was carried out to investigate the feasibility and potential importance of therapeutic monitoring following high-dose carboplatin treatment in children. High-dose carboplatin was administered over 3 or 5 days, with the initial dose based on renal function, to achieve target area under the plasma concentration–time curve (AUC) values of 21 or 20 mg ml^−1^.min, respectively. Dose adjustment was carried out based on observed individual daily AUC values, to obtain the defined target exposures. Platinum–DNA adduct levels were determined in peripheral blood leucocytes and toxicity data were obtained. Twenty-eight children were studied. Based on observed AUC values, carboplatin dose adjustment was performed in 75% (21 out of 28) patients. Therapeutic monitoring resulted in the achievement of carboplatin exposures within 80–126% of target AUC values, as compared to estimated exposures of 65–213% of target values without dose adjustment. The carboplatin AUC predicted with no dose modification was positively correlated with pretreatment glomerular filtration rate (GFR) values. Higher GFR values were observed in those patients who would have experienced AUC values >25% above the target AUC than those patients attaining AUC values >25% below the target AUC, following renal function-based dosing. Platinum–DNA adduct levels correlated with observed AUC values on day 1 of carboplatin and increased over a 5-day course of treatment. Real-time monitoring of carboplatin pharmacokinetics with adaptive dosing is both feasible and necessary for the attainment of consistent AUC values in children receiving high-dose carboplatin treatment. Pharmacodynamic data suggest a strong correlation between carboplatin pharmacokinetics and the drug–target interaction.

Carboplatin is a second-generation platinum compound commonly used in paediatric oncology and currently plays a key role in the treatment of many tumours, including neuroblastoma, rhabdomyosarcoma, brain tumours and germ cell tumours (Gaynon, 1994). It is estimated that approximately one-third of children with solid tumours will receive carboplatin as part of multimodal chemotherapy at some point during their treatment. As the pharmacokinetics of carboplatin are largely determined by the renal function of the patient being treated, dosing formulae have been devised to calculate the dose of carboplatin required to achieve the desired target exposure or area under the plasma concentration–time curve (AUC) (Calvert *et al*, 1989; Newell *et al*, 1993; Ando *et al*, 2000). This approach has been shown to result in more consistent exposure to carboplatin than dosing based on body surface area in a randomized, cross-over study in children with cancer (Thomas *et al*, 2000). Renal function-based carboplatin dosing has now become widely accepted, with an increasing number of paediatric clinical protocols specifying carboplatin dosed to a target AUC. The rationale for this approach is supported by the observation that carboplatin AUC is correlated more closely than drug dose with both clinical toxicity and response in adults and children (Newell *et al*, 1987; Jodrell *et al*, 1992).

Although this approach is now routinely used for carboplatin administered at conventional doses (AUC 4–7 mg ml^−1^.min), its application to high-dose carboplatin chemotherapy is less well understood. When high-dose chemotherapy is used in the treatment of poor prognosis patients, saturation of protein binding or drug metabolism may result in unpredictable pharmacokinetic variation. Our group and others have previously suggested that real-time carboplatin monitoring, involving dose modification based on pharmacokinetic variation, can result in the achievement of target exposures in individual patients following carboplatin chemotherapy (Veal *et al*, 1999; Chatelut *et al*, 2000; Rubie *et al*, 2003). This approach to treatment may be particularly important in a high-dose chemotherapy setting where treatment-related deaths are not uncommon. The current study, involving a relatively large number of patients being treated with high-dose carboplatin, was designed to determine whether adaptive dosing would allow target AUC values to be consistently achieved, while reducing the variability in plasma concentrations as compared to renal function-based dosing.

In addition to learning more about the pharmacokinetics of drugs used in the treatment of children with cancer, it is also important to consider molecular pharmacodynamic interactions. Although a number of studies have indicated relationships between platinum–DNA adduct formation in peripheral blood leucocytes and clinical response and toxicity following cisplatin treatment, there is little evidence to support the use of pharmacodynamic end points for carboplatin (Reed *et al*, 1990; Schellens *et al*, 1996; Veal *et al*, 2001). Whether or not this can be explained by a lack of correlation between carboplatin pharmacokinetics and pharmacodynamics is at present unclear.

The design of a United Kingdom Children's Cancer Study Group (UKCCSG) protocol for the treatment of low-risk patients with metastatic rhabdomyosarcoma and other malignant soft tissue sarcomas (MMT 98 study) provided a rare opportunity to investigate carboplatin exposure, platinum–DNA adduct formation and clinical toxicity, in a single agent high-dose paediatric setting. Additional data were also obtained from a UKCCSG study involving the treatment of recurrent central nervous (CNS) system primitive neuroectodermal tumours (Recurrent PNET study). Carboplatin was administered with target AUCs of 20 mg ml^−1^.min over 5 days or 21 mg ml.min over 3 days for the treatment of soft tissue sarcoma or recurrent PNET patients respectively. Carboplatin pharmacokinetics were monitored following initial renal function-based dosing, with dose adjustments implemented based on observed AUC values. In a subset of patients, platinum–DNA adduct levels were determined in peripheral blood leucocytes.

## PATIENTS AND METHODS

### Patient eligibility and treatment

The study protocols were approved by the UK Northern and Yorkshire Multicentre Research Ethics Committee and participating centres obtained local ethical approval; written informed consent was required, either from patients or parents as appropriate, for all patients entered onto the study. Patients, 21 years or younger, receiving high-dose carboplatin chemotherapy as part of their standard clinical treatment, were eligible (Table 1). Patients were being treated on clinical protocols for either soft tissue sarcoma (UKCCSG MMT 98 study) or recurrent primitive neuroectodermal tumour (UKCCSG recurrent PNET study). All patients were required to have central venous access, in the form of double-lumen central venous catheters, to participate in this pharmacokinetic study.

Carboplatin was administered diluted in 5% dextrose, as a 60 min intravenous infusion, as part of the standard chemotherapy regimen that each patient was currently receiving. The dose of carboplatin administered on day 1 of treatment was determined by the renal function of the patient, based on either ^51^Cr-EDTA half-life or glomerular filtration rate (GFR), using equations described previously (Newell *et al*, 1993). Patients being treated for soft tissue sarcoma received carboplatin dosed to a target AUC of 20 mg ml^−1^.min over a 5-day treatment period. Before entering this high-dose consolidation phase of chemotherapy, patients were entered onto a phase II ‘window study’ consisting of single-agent carboplatin targeted to obtain an AUC of 10 mg ml^−1^.min on each of the two courses of treatment. Patients being treated for recurrent CNS primitive neuroectodermal tumours received carboplatin dosed to a target AUC of 21 mg ml^−1^.min over 3 days. These dosages were determined by the clinical protocols for the specific tumour types. Toxicity following carboplatin treatment was assessed by the National Cancer Institute Common Toxicity Criteria (CTC), version 2.0.

### Blood sampling and analysis

Blood samples (2 ml) for pharmacokinetic analysis were obtained from a central line before carboplatin infusion, 30 min after the start of infusion, at 60 min (end of infusion) and at 120 min after the start of infusion (60 min post-infusion). All samples were taken from a different lumen from that used for drug administration. Plasma was separated from whole blood samples by centrifugation (1200 g, 4°C, 10 min), and 1 ml was then removed and placed in an Amicon Centrifree micropartition unit with a 30 000 MW cutoff (Millipore, Edinburgh, UK). This plasma sample was centrifuged (1500 g, 4°C, 15 min) to obtain plasma ultrafiltrate for determination of free carboplatin levels. Samples were sent by overnight courier, on dry ice and in an insulated container, to the Northern Institute for Cancer Research, Newcastle University, and were stored at −20°C before analysis.

Platinum pharmacokinetic analyses were carried out by flameless atomic absorption spectrophotometry (AAS) using a Perkin–Elmer AAnalyst 600 graphite furnace spectrometer (Perkin-Elmer Ltd, Beaconsfield, UK). Free or unbound platinum levels were determined in plasma ultrafiltrates as described previously (Veal *et al*, 2001). All samples were analysed in duplicate and values are expressed as the average of these measurements. Duplicate values were within 15% of each other in all cases. Intra- and interassay coefficients of variation for a quality assurance sample had to be <10% for an assay to be valid. The limit of detection for the AAS assay was 0.10 *μ*g ml^−1^.

### Pharmacokinetics and dose adjustment

Carboplatin clearance and AUC were determined by Bayesian analysis following each dose of carboplatin using a two compartment model as described previously (Peng *et al*, 1995). For patients being treated on a 5-day carboplatin schedule, dosing was adjusted on days 2–5, based on drug exposures and clearance values determined for day 1, or for days 1 and 2, to achieve the desired target cumulative AUC of 20 mg ml^−1^.min. For patients being treated on the 3-day carboplatin schedule, dosing was adjusted on day 3, based on drug exposure on day 1, to achieve the desired target cumulative AUC of 21 mg ml^−1^.min. Carboplatin dose adjustments were recommended for all patients with day 1 AUC values ⩾10% outside the target daily AUC defined in the clinical protocol on which the patient was being treated. Dose adjustments were calculated based on the actual carboplatin clearance determined on day 1 and the remaining AUC to be achieved.

### Platinum–DNA adduct measurement

Whole blood samples were taken before the start of treatment, 24 h after the first dose of carboplatin on day 1 and 24 h after the final dose of carboplatin on day 5 for those patients being treated over a 5-day period. Cellular DNA was isolated from peripheral blood leucocytes as described previously (Peng *et al*, 1995) and the concentration of DNA in each sample was quantified by UV absorption (*A*_260_). DNA samples were diluted in 3.5% nitric acid and were hydrolysed overnight at 70°C. Platinum–DNA adduct levels were determined by ultrasensitive multi-collector inductively coupled plasma mass spectrometry (ICP-MS) as described previously (Cooper *et al*, 2004; Nowell *et al*, 2005). The limits of detection and quantitation using this methodology were 0.3 and 1 amol platinum ml^−1^ (1 amol=1 × 10^−18^ mol ml^−1^), respectively. Final platinum–DNA adduct levels were calculated as nmoles g^−1^ DNA and the results of duplicate analyses were within 10% of each other in all cases.

### Statistical analysis

Linear regression analysis and the Pearson correlation coefficient were used to indicate correlations between patient GFR and % target carboplatin AUC and between observed carboplatin AUC and platinum–DNA adduct levels. The unpaired two-sided Student's *t*-test was used to determine differences between GFR values in patients who would have experienced AUC values greater than 25% above the target AUC and in patients who would have experienced AUC values greater than 25% below the target AUC, if no carboplatin dose modification had been carried out. The paired Student's *t*-test was used to determine differences between carboplatin clearance values observed on day 1 *vs* day 3 and on day 1 *vs* day 5 of treatment. Log-transformed values of AUC, GFR and clearance were used for all statistical tests described.

## RESULTS

### Patient characteristics and treatment

Children were treated at 12 UKCCSG centres for soft tissue sarcoma or recurrent primitive neuroectodermal tumour. Twenty-eight children and adolescents receiving carboplatin were entered onto the two studies between September 1998 and November 2005. The study population had a mean age of 11.7 years (range 1–21) and included 14 men and 14 women. Renal function-based carboplatin dosing was carried out on day 1 of treatment, with the target exposure defined in the clinical protocol on which the child was being treated. Patient characteristics including age, sex and tumour type are given in Table 1.

### Pharmacokinetics and dose adjustment

Carboplatin clearance values ranged from 25 to 195 ml min^−1^ and showed a good correlation with GFR values of 21–228 ml min^−1^ (50–200 ml min^−1^ 1.73 m^−2^) determined before day 1 of treatment (*r*=0.73, *P*< 0.0001). No significant changes in clearance were observed in those patients where clearance values were determined on days 1 and 3 of carboplatin treatment (mean of differences: 9.6 ml min^−1^; 95% CI −2.9 to 22.1; *n*=17; *P*=0.32) or on days 1 and 5 of treatment (mean of differences: 8.5 ml min^−1^; 95% CI −4.3 to 21.3; *n*=12; *P*=0.16).

**Figure 1:**
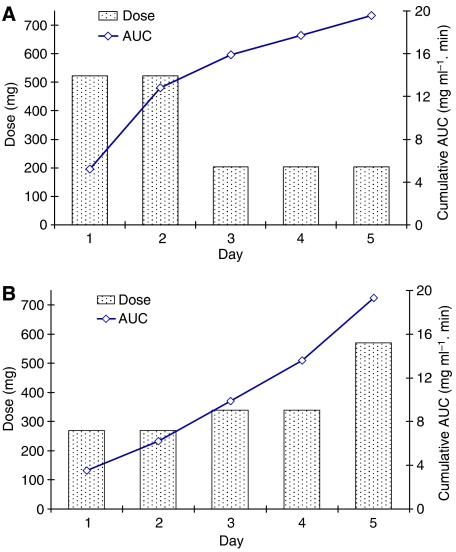
Examples of carboplatin pharmacokinetically guided dosing and exposure (AUC) in individual patients showing (**A**) a dose reduction implemented on day 3 to achieve a cumulative AUC of 19.6 *μ*g ml^−1^.min over 5 days of treatment and (**B**) dose increases on days 3 and 5 resulting in a cumulative AUC of 19.3 *μ*g ml^−1^.min.

**Figure 2:**
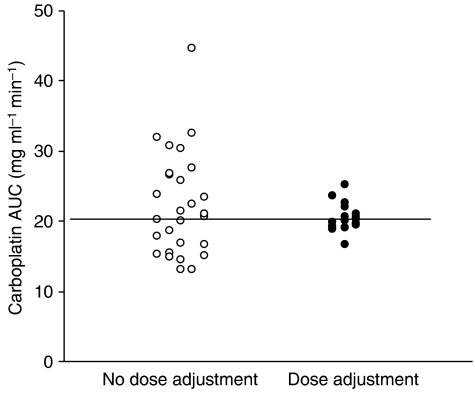
Predicted *vs* actual carboplatin exposures following pharmacokinetically guided dosage adjustment in children receiving high-dose carboplatin chemotherapy (*n*=28).

Pharmacokinetic monitoring was carried out in 28 patients receiving high-dose carboplatin chemotherapy. Details of estimated and actual total doses and carboplatin exposures are shown for all patients in Table 2. On the basis of AUC values obtained on day 1 following renal function-based dosing, and target daily AUC values defined in the treatment protocol, carboplatin dose adjustment was carried out in 21 out of 28 patients (75%). Dosage adjustments from the day 1 dose were carried out on day 2 of treatment in one patient, day 3 in 12 patients, day 4 in six patients, with two different adjustments carried out on both days 3 and 5 in the remaining two patients. Dosage adjustments in these 21 patients ranged from 7 to 67% of the initial renal function-based dose. For those patients where carboplatin dose was modified, dose increases were implemented in 43% of patients (9/21) and dose reductions in 57% (12/21). Examples of carboplatin dosing and exposure in individual patients requiring dosage adjustments based on drug exposure data are shown in Figure 1. Overall, pharmacokinetically guided dose adjustment resulted in achievement of AUC values of 16.7–25.2 mg ml^−1^.min (84–126% of target AUC values) as compared to estimated AUC values of 13.0–44.7 mg ml^−1^.min (65–213% of target AUC values) without dose adjustment (Figure 2). Estimated AUC values were calculated based on the observed daily carboplatin clearance values, assuming that the day 1 renal function-based dose had been administered on each day of treatment, as opposed to the adjusted dose actually administered.

**Figure 3:**
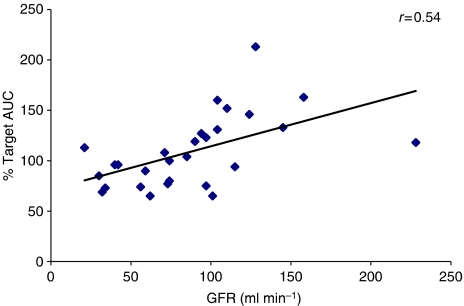
Correlation between patient renal function (GFR) and estimated % target carboplatin AUC with no pharmacological dose adjustment (*n*=28).

The relationship between the renal function of patients receiving high-dose carboplatin treatment and the predicted AUC that would have been obtained if no dose modification had been carried out is shown in Figure 3 (*r*=0.54; *P*=0.003). Pretreatment GFR values were higher in those patients who would have experienced AUC values greater than 25% above the target AUC (mean value 121 ml min^−1^, range 94–158; standardized mean value 137 ml min^−1^ 1.73 m^−2^, range 81–200; *n*=8) than those who would have experienced AUC values >25% below the target AUC (mean value 57 ml min^−1^, range 32–101; standardized mean value 81 ml min^−1^ 1.73 m^−2^, range 50–111; *n*=5) (*P*=0.0139).

### Platinum–DNA adduct measurements

**Figure 4:**
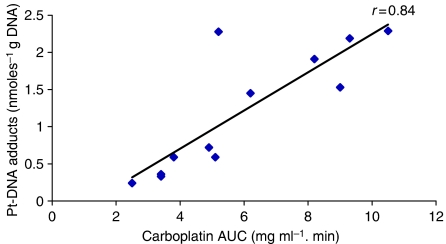
Correlation between actual carboplatin exposure (AUC) and platinum–DNA adduct levels measured in peripheral blood leucocytes obtained 24 h after high-dose carboplatin administration on day 1 of a course of treatment. Data represents samples analysed from six patients receiving carboplatin on day 1 of high-dose carboplatin treatment (MMT 98 study) and three patients receiving carboplatin on day 1 of each of two courses of a high-dose carboplatin window study (MMT 98).

Carboplatin–DNA adduct levels were measured in DNA samples isolated from peripheral blood leucocytes from 8 of the 17 patients on the MMT 98 study on a total of 12 courses of carboplatin administration. Samples were obtained following carboplatin dosed to a target AUC of 4 mg ml^−1^.min on day 1 of a 5-day course of treatment in six patients (carboplatin dose range: 380–957 mg), following carboplatin on day 5 of treatment in four of these patients (carboplatin dose range: 203–860 mg) and following a single dose of carboplatin targeted to an AUC of 10 mg ml^−1^.min as part of a phase II window study in a total of three patients (carboplatin dose range: 1140–1550 mg). Platinum–DNA adduct levels determined 24 h post-administration on day 1 of treatment ranged from 0.24 to 2.29 nmoles g^−1^ DNA and correlated with the observed carboplatin AUC values as shown in Figure 4 (*r*=0.84, *P*=0.0006 for all data points shown; *r*=0.81, *P*=0.01 when the analysis is limited to a single data point for each individual patient). Additional measurements determined 24 h post-administration on day 5 of treatment were obtained for four patients and indicated a clear increase in platinum–DNA adduct levels as compared to the corresponding day 1 levels (*P*=0.01), suggesting an increased level of adduct formation or an accumulation of adducts over the 5-day treatment period (Table 3). Overall, platinum–DNA adduct levels ranged from 0.24 to 6.99 nmole g^−1^ DNA, with corresponding peak unbound carboplatin plasma concentrations ranging from 15.8 to 76.6 *μ*g ml^−1^. These levels are well above the limits of detection and quantitation of the ICP-MS technique used (16). No correlations were observed between platinum–DNA adduct formation and other parameters investigated, such as body weight, age or gender of patients studied.

### Carboplatin toxicity

Toxicity data were available for a total of 23 of the 28 patients studied, including 14 patients treated on the MMT 98 study and nine patients on the PNET study. Haematological toxicity (CTC grade 3 or 4), including neutropenia and thrombocytopenia, was observed in 91% (21/23) of patients overall. This level of haematological toxicity was reported in 86% of MMT 98 patients (12/14), who received carboplatin targeted to an AUC of 20 mg ml^−1^.min over a 5-day treatment period, and in 100% of patients (9/9), who received high-dose carboplatin targeted to an AUC of 21 mg ml^−1^.min over the shorter 3 day regimen on the PNET study. No treatment-related deaths were observed in any of the 28 patients studied.

## DISCUSSION

The current study was carried out to investigate the variation in carboplatin pharmacokinetics and exposure and to determine the potential importance of therapeutic monitoring following high-dose carboplatin treatment in children. In addition, platinum–DNA adduct levels were measured in peripheral blood leucocytes, to allow a comparison of carboplatin pharmacodynamics and pharmacokinetics alongside clinical toxicity data. Clinical response data were not evaluated due to the potential role played by concomitant chemotherapy, administered either before or following high-dose carboplatin treatment, on the tumour responses observed.

A total of 28 patients were included in the study with the dose of carboplatin on day 1 based on renal function to achieve a target AUC. No significant interoccasion variability in carboplatin clearance was observed in those patients where clearance values were determined on the first and last days of treatment, although mean clearance values were lower on days 3 and 5 *vs* comparable day 1 data. Interestingly, these data are in agreement with those published from a phase I study, which reported a statistically significant, but limited, decrease in clearance between days 1 and 5 of high-dose carboplatin treatment (Rubie *et al*, 2003).

On the basis of the observed AUC values in individual patients, carboplatin dose adjustment was carried out in 75% of patients, with a range of dose adjustments up to a maximum of 67% from the initial estimated dose. These dose changes resulted in drug exposures of 84–126% of the target AUC values as defined in the two study protocols. In comparison, estimated carboplatin exposures of 65–213% of the target AUC values would have been attained without dose adjustment, with 32% of patients (9/28) receiving carboplatin AUC values above 25 mg ml^−1^.min. The likelihood of dose adjustment was not influenced by the method of GFR determination or the dosing equation used to determine initial renal function-based dosing. Based on previous studies showing correlations between carboplatin AUC and drug toxicity following high-dose chemotherapy, it is likely that patients with an AUC greater than 25 mg ml^−1^.min would have experienced serious side effects if therapeutic monitoring had not been implemented (Huitema *et al*, 2002; Kloft *et al*, 2002). Indeed, significantly increased frequencies of nephrotoxicity, ototoxicity and peripheral nervous system toxicities were observed in patients with AUC above 24.2 mg ml^−1^.min in a recently published study in patients with germ-cell cancer (Kloft *et al*, 2003).

In the current study, a positive correlation was observed between renal function (GFR) and the estimated AUC that would have been observed if pharmacologically guided dosing had not been performed. Pretreatment GFR values were significantly higher in those patients whose AUC values would have exceeded the target by 25% without dose modification (*n*=8), than in those whose AUC values would have been more than 25% below the target AUC (*n*=5). There was a greater than twofold difference in mean GFR between these two groups (*P*=0.0139). These data suggest that the current use of dosing equations is less effective when dealing with patients receiving high-dose carboplatin chemotherapy, with children with higher GFR values being more likely to attain AUC values greater than intended, and those with lower GFR values being at a greater risk of under-exposure.

Platinum–DNA adducts were measured at 24 h after carboplatin administration, as this has previously been shown to be the time when peak adduct levels are observed following platinum drug treatment (Peng *et al*, 1997). In addition, this allowed the blood sample to be drawn immediately before the next dose of carboplatin being administered in those patients receiving the drug over several days. A strong correlation was observed between the formation of platinum–DNA adducts and carboplatin AUC on day 1 of treatment, with higher adduct levels being observed in peripheral blood leucocytes obtained from patients with higher plasma carboplatin concentrations. Pharmacokinetic–pharmacodynamic comparisons with the platinum drugs have not always shown a consistent positive relationship (Schellens *et al*, 1996; Peng *et al*, 1997; Veal *et al*, 2001). However, carboplatin seems to show a stronger trend towards such a relationship than does cisplatin (Ghazal-Aswad *et al*, 1999). The correlation between this pharmacodynamic measure and drug exposure supports the use of dosing based on renal function and adaptive dosing to achieve target carboplatin AUC values. Similarly, the lack of a pharmacokinetic–pharmacodynamic relationship for cisplatin may explain why such an approach to dosing has not been established for this drug.

The current study also suggests that when carboplatin is administered over several days, there is either an increased formation of platinum–DNA adducts or an accumulation of adducts over the treatment period. Despite the complication of carboplatin dose variations between days 1 and 5 of treatment, owing to pharmacokinetic dose adjustment, adduct levels following carboplatin administration on day 5 were between 3- and 10-fold higher than the corresponding levels following day 1 of the treatment. This phenomenon may explain some of the apparent inconsistencies in published pharmacokinetic–pharmacodynamic relationships for this drug. For example, although a strong correlation was observed between platinum–DNA adduct levels and carboplatin systemic exposure following administration on day 1 in the current study, the accumulation of adduct levels observed following several days of treatment negated this relationship when data from all days of treatment were included in a similar analysis. Although we are unable to determine cumulative platinum–DNA adduct levels over the 5-day treatment period, with no information on adduct levels on days 2–4, it is possible that differences in total adduct levels may reflect differences in observed toxicity among some patients, despite comparable cumulative pharmacokinetic exposures in terms of AUC. Indeed, correlations have previously been shown between platinum–DNA adduct levels and leukocytopenia following cisplatin treatment (Veal *et al*, 2001).

Haematological toxicity was observed in the vast majority of patients studied (90%), as anticipated with the use of high-dose carboplatin chemotherapy. The high percentage of patients experiencing CTC grade 3 and 4 haematological toxicity is likely to be associated with the high carboplatin exposures achieved in all patients through adaptive dosing. Estimated exposures as low as 13.0 mg ml^−1^.min, which would have been achieved in some of the patients studied if dose adjustment had not been carried out, may not have led to the same grade toxicity but may also have been less likely to have resulted in clinical responses. Correlations between decreased carboplatin AUC values and increased rates of relapse have previously been reported in patients with testicular germ-cell tumours (Horwich *et al*, 1991). Although an evaluation of clinical response data was not included in this study, owing to the potential influence of concomitant chemotherapy, it is encouraging that no treatment-related deaths were observed in any of the patients on the current study. This is particularly the case in light of the high incidence of treatment-related deaths previously reported with the use of high-dose carboplatin in paediatric patients (Santana *et al*, 1992; Jakacki *et al*, 1997; Dunkel *et al*, 1998).

Data from this multi-centre study show the feasibility of real-time monitoring of carboplatin pharmacokinetics with adaptive dosing and indicate that this approach is necessary for the attainment of consistent AUC values in individual patients receiving high-dose carboplatin treatment. This approach is now being used in clinical studies in the UK, with the aim of improving efficacy and minimising toxicity of carboplatin in similar high-dose protocols, and is likely to be relevant to the treatment of both childhood and adult cancer patients. The pharmacodynamic data presented here suggest that a strong correlation exists between the pharmacokinetics of carboplatin and the drug–target interaction.

## Figures and Tables

**Table 1:** Patient characteristics

**Characteristic**	**No. of patients**	**%**
*Age, (years)*		
<5	4	14
5–9	5	18
10–14	12	43
15–21	7	25
		
*Sex*		
Male	14	50
Female	14	50
		
*Diagnosis*		
Soft tissue sarcoma (MMT 98)	5	18
Rhabdomyosarcoma (MMT 98)	12	43
PNET	11	39
		
*Additional chemotherapy*		
Etoposide	17	61
Cyclophosphamide	28	100
Thiotepa	11	39

PNET, primitive neuroectodermal tumour.

For patients treated on the MMT 98 study, cyclophosphamide (2 g m^−2^ day^−1^ × 3) was administered to patients on two courses of treatment, 6 and 2 weeks before receiving high-dose carboplatin, with etoposide (800 mg m^−2^ day^−1^ × 3) given 4 weeks before carboplatin. Patients treated on the PNET study received both cyclophosphamide (2 g m^−2^ day^−1^ × 2) and thiotepa (300 mg m^−2^ day^−1^) before high-dose carboplatin with time between chemotherapy treatments dependent on neutrophil and platelet count recovery.

**Table 2:** Carboplatin doses and estimated renal function-based dosing AUC *vs* actual AUC following pharmacologically guided dosing

							**Estimated**		**Achieved**	
**Study**	**Patient**	**GFR (ml min^−1^)**	**Target AUC (mg ml^−1^. min)**	**Dose modification**	**Estimated total dose (mg)^a^**	**Actual total dose (mg)**	**AUC^b^ (mg ml^−1^.min)**	**% target AUC**	**AUC (mg ml^−1^.min)**	**% Target AUC**
MMT 98	1	110	20	Day 3	2610	1653	30.3	152	19.6	98
	2	115	20	Day 3	2720	2921	18.7	94	ND	ND
	3	104	20	Day 3	2350	1450	32.0	160	19.8	99
	4	158	20	Day 2	3660	2868	32.6	163	25.2	126
	5	85	20	None	2090	2090	20.7	104	20.7	104
	6	71	20	None	1750	1750	21.5	108	ND	ND
	7	73	20	Day 4	1900	2860	15.4	77	23.7	119
	8	90	20	Day 4	1565	1243	23.8	119	19.0	95
	9	21	20	Day 3	575	508	22.5	113	ND	ND
	10	228	20	Day 3	4785	4071	23.5	118	ND	ND
	11	30	20	Day 3	750	970	16.9	85	22.1	111
	12	56	20	Days 3/5	1350	1790	14.8	74	19.3	97
	13	59	20	Days 3/5	1475	1575	17.9	90	19.5	98
	14	62	20	Day 4	1500	2500	13.0	65	22.6	113
	15	97	20	Day 4	2160	2696	15.0	75	18.8	94
	16	101	20	Day 4	2650	4070	13.0	65	ND	ND
	17	145	20	Day 4	3400	2664	26.5	133	20.1	101
PNET	18	34	21	None	1026	1026	15.3	73	ND	ND
	19	104	21	Day 3	3720	2830	27.6	131	ND	ND
	20	74	21	None	1590	1590	16.7	80	16.7	80
	21	97	21	Day 3	3141	2554	25.8	123	ND	ND
	22	124	21	Day 3	3258	2387	30.7	146	20.7	99
	23	32	21	Day 3	900	1130	14.5	69	19.0	90
	24	128	21	Day 3	4320	2880	44.7	213	ND	ND
	25	74	21	None	1608	1608	21.0	100	21.0	100
	26	42	21	None	1020	1020	20.1	96	20.1	96
	27	94	21	Day 3	3000	2360	26.7	127	ND	ND
	28	40	21	None	870	870	20.2	96	20.2	96
										
					Range:	MMT 98	13.0–32.6	65–163	18.8–25.2	94–126
						PNET	14.5–44.7	69-213	16.7–21.0	80–100

a Estimated total dose is based on the day 1 renal function-based dose without modification.

b Estimated total AUC is that which would have resulted if no dose adjustment had been made (based on daily carboplatin clearance values)

**Table 3:** Platinum–DNA adduct levels determined in patients following carboplatin administration on days 1 and 5 of treatment in four patients on the MMT 98 protocol

**Patient**	**Study day**	**Carboplatin dose (mg)**	**Carboplatin AUC (mg ml^−1^** **.min)**	**Pt-DNA adducts (nmol g^−1^DNA)**
1	1	522	5.2	2.28
	5	203	1.9	6.99
2	1	380	2.5	0.24
	5	860	7.8	2.21
3	1	732	5.1	0.59
	5	534	5.8	4.06
4	1	418	3.8	0.57
	5	418	4.2	3.21
